# The Role of Extracellular Vesicles from Human Macrophages on Host-Pathogen Interaction

**DOI:** 10.3390/ijms221910262

**Published:** 2021-09-23

**Authors:** Luis A. Arteaga-Blanco, Dumith Chequer Bou-Habib

**Affiliations:** 1 Laboratory on Thymus Research, Oswaldo Cruz Institute/Fiocruz, Rio de Janeiro 21040-900, Brazil; 2 National Institute of Science and Technology on Neuroimmunomodulation, Rio de Janeiro 21040-900, Brazil

**Keywords:** extracellular vesicles, human macrophages, innate immunity, host–pathogen interaction and infectious diseases

## Abstract

The nano-sized membrane enclosed extracellular vesicles (EVs) released by virtually all cell types play an essential role in intercellular communication via delivering bio-molecules, such as nucleic acids, proteins, lipids, and other molecules to recipient cells. By mediating an active and steady-state cell-to-cell communication, EVs contribute to regulating and preserving cellular homeostasis. On the other hand, EVs can also spread pathogen-derived molecules during infections, subverting the host immune responses during infections and thus worsening pathophysiological processes. In recent years, the biological functioning of EVs has become a widespread research field in basic and clinical branches of medical sciences due to their potential role in therapeutic applications for several diseases. This review aims to summarize the main recent findings regarding the implication of EVs shed by human macrophages (MΦ-EVs) and how they can modulate the host immune response to control or increase the damage caused by infectious agents. We will also present the methods used to describe MΦ-EVs, as well as the potential of these EVs as disease diagnostic tools for some human pathogens. We believe that an in-depth understanding of the host–pathogen interactions mediated by MΦ-EVs may trigger the development of innovative therapeutic strategies against infectious diseases.

## 1. Introduction

**Figure 1:**
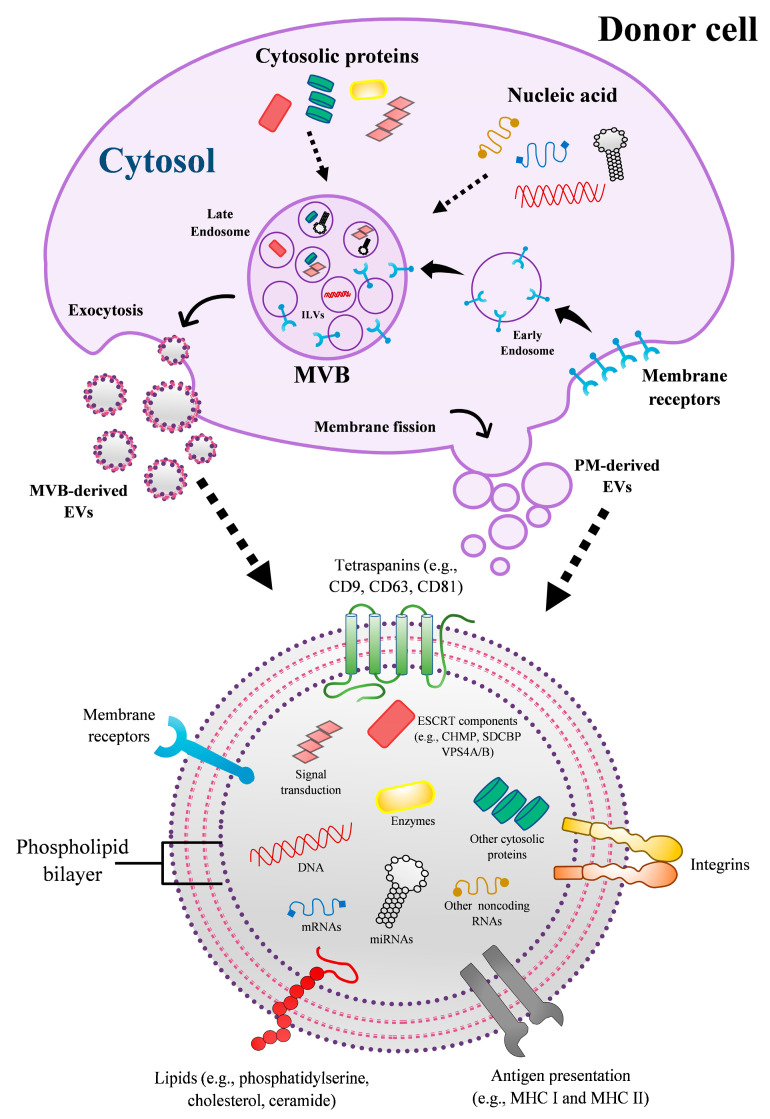
Schematic representation of origin and composition of EVs secreted by eukaryotic cell. PM-derived EVs originate directly from the cell membrane fission, whereas EVs of endosomal system originate from ILVs contained in cytosolic MVBs, which later fuse with the PM releasing the vesicles in the outer space. EVs can carry proteins, lipids, and nucleic acids. Some of the listed components represented in the figure may be enriched in some subtypes of EVs and not in others. For instance, ESCRTs components (e.g., CHMP, SDCBP, VPS4A/B) are highly enriched in MVB-derived EVs rather than PM-derived EVs. Abbreviations: CHMP, charged multivesicular body protein; ESCRT, endosomal sorting complex required for transport; ILVs, intraluminal vesicles; MVBs, multivesicular bodies; MHC, major histocompatibility complex; PM, plasma membrane; SDCBP, syndecan binding protein; VPS4A/B, vacuolar protein sorting-associated protein 4A/B.

Many years later, facing the vast progress in understanding the macrophage role in homeostasis, researchers turn their minds to that remote sunny afternoon by the sea, when Elie Metchnikoff described and portrayed these cells for the first time. Macrophages were then defined only by their ability to engulf foreign bodies (see Acknowledgments). Today, we know that in addition to being involved in host defense and other biological functions, macrophages participate in cellular communication through extracellular vesicles (EVs), which are small lipid bilayer-enclosed structures released from cells in the extracellular space [1,2]. EVs are implicated in intercellular communication without direct cell-to-cell contact trough the transport and transfer of bioactive molecules such as nucleic acids, proteins, lipids, metabolites, cytokines, chemokines, among other molecules, to recipient cells [3,4] (Figure 1). All these components are further processed to promote physiological or pathological alterations in recipient cells, including modulation of the effector activities of cells of the immune system [4,5]. Although there has been much progress in recent years on the understanding of the fundamental biology of EVs, some aspects related to their biogenesis, secretion mechanisms, interaction with the recipient cell, and biological function remains unclear [4,6]. Therefore, more research focused on the comprehension of the mechanisms that mediate the biological effects of EVs on their cellular targets under specific physiological or pathological condition could clarify open questions in the field of extracellular vesicles, including EV diversity, composition, pathophysiology activities, and their role on host–pathogen interactions.

Different types or subtypes of vesicles can be secreted by the same cell. To date, the biosynthesis pathways (vesicles origin) and biophysical and biochemical characteristics (size, shape, densities, biochemical markers, protein, and lipid composition) are features commonly used to classify EVs [1,3]. For instance, based on size and origin, vesicles can be categorized into large EVs (LEVs, >300 nm in diameter), released by the fission of the plasma membrane from apoptotic or healthy cells [1,7]; intermediate EVs (IEVs, 150–300 nm), and small EVs (sEVs, <150 nm), these latter types originated from plasma membrane or endosomal system [6,8]. sEVs from the endosomal system originate from intraluminal vesicles (ILVs) contained in cytosolic multivesicular bodies (MVBs), which later fuse with the plasma membrane releasing the vesicles in the outer space [9,10] (Figure 1).

Many studies have shown multiple ways by which several pathogens modify EV synthesis, enhancing or restricting their replication and dissemination in receptor cells [11,12,13]. In fact, there is accumulating evidence of the release of vesicles from several sources during infectious diseases, acting both in pathogen–pathogen inter-communication as well as in host–pathogen interaction [11,14,15,16,17,18,19]. However, despite the increasing number of reviews describing and discussing the involvement and role of EVs in the complex scenario of host–pathogen interaction, up-to-date information about the specific impact of vesicles released by human macrophages on the progression of infectious illnesses is quite limited.

Macrophages are key phagocytic mononuclear cells of the myeloid lineage distributed in different organs (e.g., liver, lungs, skin, heart), and every tissue of the immune system, such as lymph nodes, spleen, and bone marrow [20,21,22]. Macrophages are implicated in multiple activities essential for bone remodeling, tissue development and homeostasis, resolution of inflammation, and wound healing. In addition, these cells induce and orchestrate the innate and acquired immunity against infectious agents through a variety of cell processes, such as antigen presentation, secretion of pro- and anti-inflammatory cytokines, chemokines, and production of antibacterial peptides and antiviral compounds (e.g., IL-6, IL-1β, LL-37, reactive oxygen, and nitrogen species) [23,24,25]. Given their ability to recognize and respond to a wide range of stimuli and pathogens through phagocytic activity and secretion of soluble mediators, which may favor or inhibit the survival and persistence of infectious agents, understanding the intricate and complex roles of macrophages in host–pathogen interaction is a challenging task, whichrequires permanent attention of investigators to new concepts often arising in this field of life science [26,27].

This review aims to summarize principal findings regarding the implication of EVs released by human macrophages (MΦ-EVs) during bacterial, viral, fungal, and parasitic infection, emphasizing their role in host–pathogen interaction and their functional impacts on the host’s immune response. We will also present an overview of the isolation and characterization methods to describe vesicles from human macrophages and discuss their potential use as diagnostic tools for human infectious diseases. Highlighting the contribution of EVs to the ability of macrophages to regulate pathogen growth and propagation can support the comprehension of how cellular communication is critical to developing strategies to attenuate cellular and tissue damage caused by infectious agents.

Given the growing scientific interest in the role played by EVs in cellular communication and the undisputed importance of macrophages in the generation of an effective immune response and preservation of the homeostasis during infections by all types of pathogens, this review will encompass the more recent advances regarding EVs released from human macrophages and their ability to regulate the growth and dissemination of infectious agents.

## 2. Immunoregulatory Properties of Macrophage-Derived EVs

Numerous studies have widely documented the ability of EVs to regulate immune responses, mainly in the context of cancer and autoimmunity [28,29,30]. Cells of the immune system, such as macrophages, monocytes, neutrophils, dendritic cells (DCs), Natural Killer cells, T and B lymphocytes can shed EVs with specific cargo, resembling their origin and function [31,32], thus participating in cellular communication independently of cell–cell contact or soluble factors (e.g., cytokines and chemokines) [33]. In recent years, some authors have unveiled the emerging role of vesicles secreted by human macrophages on the direct or indirect regulation of innate and adaptive immunity [34,35,36]. As such, early studies showed that EVs released by circulating human monocyte-derived macrophages (MDM) induced proliferation and activation of CD4+ and CD8+ T cells in vitro and in vivo after intranasal injection in mice. This EV-mediated stimulus was depended on DCs activity and resulted in the induction of effector memory T cells [37]. Furthermore, macrophage-derived vesicles can carry MHC class II and costimulatory molecules, similarly to DC-derived EVs, suggesting a role in antigen presentation [36,38]. In addition, MΦ-EVs have also been shown to induce the differentiation of human monocytic THP-1 cells into macrophages through the transfer of miR-223, which is an essential regulator of myeloid cell proliferation and differentiation [34]. An interesting functional opposite effect has emerged from studies with vesicles derived from differentially activated macrophages. As such, EVs derived from pro-inflammatory macrophages induce the secretion of Th1 cell-promoting cytokines (IL-12 and IFN-γ) in both macrophages and DC cell lines and elicit a more robust antigen specific cytotoxic T cell response in vivo when administered together with a peptide vaccine. In contrast, EVs released by anti-inflammatory macrophages enhanced the secretion of the anti-inflammatory cytokines IL-4 and IL-10 by macrophages and DCs [35]. These and other studies indicate that MΦ-EVs can regulate inflammatory reactions and modulate innate immune responses, with repercussions in the outcome of infectious diseases, either by inhibiting or favoring pathogen replication, therefore ameliorating or worsening infectious illnesses. In the following sections, we will summarize and discuss the effects of human macrophage-derived EVs on host–pathogen interactions.

## 3. Human Macrophage-Derived EVs and Bacterial Infections

Many reports have described that during a bacterial invasion, vesicles secreted by infected macrophages may induce specific biological functions in recipient cell, such as enhancement of microbial survival and dissemination, or restriction and blockade of bacterial replication [12,39]. For instance, Volgers and colleagues (2017) reported that EV released by THP-1 macrophage-like cells infected with common respiratory pathogens *Haemophilus influenzae*, *Moraxella catarrhalis*, *Streptococcus pneumoniae*, or *Pseudomonas aeruginosa*, or with bacterial-derived outer membrane vesicles (OMV), elicit strong release of pro-inflammatory mediators (TNF-α, IL-8, and IL-1β) by naïve recipient macrophages. In addition, MΦ-EVs were also found to enhance bacterial adherence and increase the number of intracellular bacteria [40]. These and other findings quoted in the following sections show that EVs secreted by infected macrophages may play a dual role during bacterial infection, either dampening the host response, thereby contributing to microbial survival, or stimulating the immune system, thus favoring the restriction of pathogen replication (Table 1).

### 3.1. EVs from Macrophages Can Potentiate Bacterial Infections

Studies have shown that EVs bearing bacterial components stimulate the host immune response to promote pathogen invasion, replication, and survival in recipient cells [12,39]. For example, EVs from *Escherichia coli*-infected THP-1 cells and E. coli-infected T84 intestinal epithelial cells (IECs) can increase bacterial replication in naïve macrophages by stimulating the secretion of pro-inflammatory cytokines (TNF-α and IL-6) via nuclear factor-kappa B (NF-kB) and MAPK pathways. In contrast, EVs derived from uninfected THP-1 can reduce E. coli intracellular replication. These findings suggest that vesicles, upon altering the functioning of immune cells, can either favor or inhibit bacterial replication [41]. Moreover, circulating human monocyte-derived macrophages release EVs packing a set of miRNAs (miRs-1224, -1293, -425, -4467, -4732, -484, -5094, -6848, -6849, -96, and -4488) involved in cell metabolism pathways (e.g., carbon, amino acid, fatty acids, sugar metabolism) upon infection with *Mycobacterium bovis* Bacillus Calmette–Guerin (BCG), which may reprogram the metabolic pathways and modulate immune surveillance to allow bacterial invasion and survival in recipient BCG-infected macrophages [42]. EVs from infected macrophages can also modulate the functionality of endothelial cells, as vesicles released by *Treponema pallidum*-infected THP-1 cells affected the adhesion and permeability of human umbilical vein endothelial cells (HUVECs) through upregulating the expression of the intercellular cell adhesion molecule 1 (ICAM-1), vascular cell adhesion molecule (VCAM-1), vascular endothelial growth factor (VEGF) and IL-8 [43]. Later, the same group showed that vesicles from T. pallidum-infected THP-1 cells transferred to vascular endothelial cells (VECs) the miR-146a-5p involved in the reduction of the monocyte transendothelial migration and endothelial permeability as a result of decreased expression of junctional adhesion molecule C (JAM-C), leading to bacterial evasion from the immune response in the EVs recipient cells [44]. Pathogens can also induce changes in EV protein composition, as particles secreted by *Mycobacterium tuberculosis* (Mtb)-infected THP-1 cells packaged more membrane associated proteins (>60%) than EVs from uninfected cells [45]. Most of the proteins found in EVs from Mtb-infected cells are involved in metabolic processes, binding, and immune response [70]. Therefore, *M. tuberculosis* may use EVs shed by infected cells as an alternative strategy to ensure a microbial-favored host–pathogen interaction in nearby EVs recipient infected cells. 

### 3.2. Macrophage-EVs Can Contribute to Development of Immune Responses against Bacterial Infections

On the other hand, EVs can also function as antigen carriers to promote host response against bacterial infections [12,39]. Most of the available studies showing the role of MΦ-EVs in inducing anti-bacterial responses were carried out with Mycobacterium sp, a genus with a significant worldwide impact on public health [71,72]. However, other intracellular bacteria with the ability to modify EV cargo to regulate both innate and adaptive immune responses have also been a matter of investigations, most of them using THP-1 cells as a donor of EVs (Table 1). Bhatnagar et al. 2007 showed that vesicles released from macrophages infected with *M. tuberculosis*, *M. bovis* BCG or *Salmonella typhimurium* transport pathogen-associated molecular patterns (PAMPs), which can stimulate pro-inflammatory responses in recipient naïve macrophages via Toll-like receptors (TLRs) and myeloid differentiation factor 88 (MyD88) pathways [46]. Similarly, EVs derived from mycobacteria-infected macrophages carrying mycobacterial antigens activate *M. tuberculosis*-specific CD4+ T cells in vivo and in vitro in a dendritic cell-dependent manner. In addition, *M. tuberculosis*-infected mice injected with EVs from *M. tuberculosis*-infected macrophages showed heightened production of TNF-α and recruitment of immune cells to the infection site to control bacterial replication [47]. Hare and colleagues (2015) described that *M. tuberculosis* infection increased the EV packaging of proteins with immune functions, including interferon-stimulated gene (ISG)-15 and interferon induced protein with tetratricopeptide repeats (IFIT)-1, -2, and -3 [48]. Furthermore, EVs from *M. tuberculosis*-infected cells increased in recipient uninfected THP-1 cells, the gene expression of ISG15, IFIT1, -2, -3, Interferon-induced transmembrane protein (IFITM) -1/3, and the secretion of pro-inflammatory cytokines IL-8, Interferon gamma inducible protein 10 (IP-10) and macrophage inflammatory protein-1 Alpha (MIP-1α) [48]. Studies conducted by Wang and colleagues (2014) showed that both *Mycobacterium avium* sp. Paratuberculosis and particles shed from uninfected macrophages enhanced the expression of surface molecules CD80 and CD86 (required for T lymphocyte activation) and stimulate the secretion of TNF-α and IFN-γ in recipient macrophages, leading to immune activation [49,73]. In addition, uninfected macrophages exposed with EVs from *M. avium*-infected macrophages increased the expression of CD40, CD80, CD81, CD86, HLA-DR, and CD195, and enhanced the secretion of IL-6, IL-8, IL-10, IFN-γ, and TNF-α [50]. Recent studies demonstrated that *M. bovis*-infected or PAMP-stimulated THP-1 cells secrete EVs packaging 5´-tRNA halves, which in turn induce innate immune responses via endosomal TLR-7 activation in recipient THP-1 cells [51]. Other examples of the ability of EVs released by macrophages to modulate the immune response against pathogens come from macrophages infected with *Legionella pneumophila* or L. pneumophila-derived outer membrane vesicles (OMVs), whose EVs stimulate the secretion of several pro-inflammatory mediators (e.g., IL-1β, IFN-β, MCP-1, TNF-α) in bystander macrophages via TLR-2 activation. The cytokines and chemokines produced by recipient macrophages may additionally activate epithelial cells, further enhancing the secretion of the chemokine CXCL8 and the recruitment of alveolar macrophages and neutrophils, eventually leading to inhibition of bacterial growth in vivo [52]. Similar control of bacterial replication was found when naïve macrophages were exposed to LPS-loaded EVs released by macrophages infected with *Salmonella enterica* serovar Typhimurium, mediated by enhanced cellular secretion of TNF-α and IL-1β via TLR-4 signaling pathway [53].

## 4. EVs from Human Macrophages and Viral Infections

Many reports have demonstrated that vesicles of several sources can play important roles during viral infections, leading to both stimulatory and inhibitory activities [13,39]. Most of the available studies published on EVs were performed with HIV-1 infection, either in vitro or ex vivo [74,75]. Because comprehensive reviews of the effect of EVs from different cellular models on viral infections were recently published elsewhere [13,74,75,76,77,78,79,80], we will focus here on the current knowledge of the pro- and anti-viral functions reported for MΦ-EVs (Table 1).

### 4.1. Stimulatory Role of Macrophage-Derived EVs during Viral Infections

Several authors reported that some viruses may exploit the endosomal machinery that produces extracellular vesicles for viral particle formation [81,82]. In this sense, EVs shed by infected cells from different sources carry viral particles or viral components (e.g., proteins and nucleic acids) that can impair host defense to promote viral infectivity and propagation [13,79,83]. For example, the transmission of human immunodeficiency viruses 1 (HIV-1) to neighboring uninfected macrophages is favored by MΦ-EVs loading viral components, an infectious process facilitated by a range of cell surface receptors and adhesion molecules [54]. Similarly, vesicles from HIV-infected macrophages containing the HIV-1 Nef protein, together with TNF-α and ADAM17, can activate latent HIV-1 infections in primary CD4+ T lymphocytes and macrophages, thus promoting viral infectivity mediated by MΦ-EVs [55]. THP-1 or primary human alveolar macrophages infected with HIV-1 can produce EVs loaded with virus-derived miRNAs (vmiR88 and vmiR99) that, when internalized by uninfected macrophages, stimulate a TLR8-dependent signaling pathway, resulting in TNF-α release [56]. These data suggest a potential role for HIV-1-derived vmiRNAs released from infected macrophages to influence viral pathogenesis. In fact, some authors reported that HIV-1 infection increased the secretion of macrophage-EVs packing miRNAs, which may play a role in viral infectivity and dissemination [57,84]. In addition, EVs loaded with miRNAs miR-23a and miR-27a secreted by Tat- and gp120-treated macrophages, harm the epithelial barrier integrity and altered mitochondrial activity, respectively, when delivered to alveolar epithelial cells [58]. These findings suggest that miRNAs carried by vesicles from HIV-1-infected macrophages may modulate the alveolar microenvironment to increase susceptibility to lung infection and injury, thus facilitating HIV-1 invasion and propagation. 

Evidence has also pointed to the fact that MΦ-EVs secreted during HIV-1 infection can trigger secondary diseases [79]. For example, HIV-1-infected macrophages promote pulmonary smooth muscle proliferation through EVs delivering pro-survival miRNA-130a, which may play role in the development of pulmonary arterial hypertension [59]. Therefore, EVs released by HIV-1-infected macrophages could participate in the dysfunction of HIV-1 non-permissive vascular cells and be involved in the development of a pulmonary disorder. Moreover, EVs released from HIV-1-infected macrophages can transfer cathepsin B (CATB) and serum amyloid p component (SAPC) to neurons, inducing neuronal apoptosis. Pre-treatment with anti-CATB and -SAPC antibodies decreased the cleavage of caspase-3 and restored neurons functionality [60], suggesting that both proteins loaded in macrophage-derived EVs may represent a therapeutic target against HIV-associated neurocognitive disorders. Finally, macrophages infected with H1N1 release vesicles containing miR-451, which attenuate, in EV recipient macrophages and DCs, the expression of IFN-β and IL-6 induced by formalin-inactivated whole-virus vaccine through the inhibition of proteins FOXO3 and ZFP36 [61,85,86], suggesting that EVs loaded with miRNAs can interfere with the innate immune response to inactivated whole virus.

### 4.2. Inhibitory Effects of Macrophage-Derived EVs during Viral Infections

Another set of studies have reported the opposite effect of MΦ-EVs on viral infection, showing that they can act as inhibitors of viral transmission and replication. For instance, U937 macrophage cell line infected with Dengue Virus 2 (DENV-2) release EVs packed with viral NS3 protein and different miRNAs, which induce changes in the expression of VE-cadherin and ICAM and increase the production TNF-α, IP-10, IL-10, RANTES, and MCP-1 in endothelial cells, resulting in protection during early stages of DENV infection [62]. Similarly, EVs secreted by macrophage infected with Hepatitis B virus (HBV) deliver IFN-induced antiviral products to hepatocytes through the T-cell immunoglobulin and mucin domain 1 (TIM-1), a receptor used by Hepatitis A Virus (HAV), indicating that anti-HBV effector molecules can be transferred by macrophage-derived EVs [63]. Moreover, EVs secreted by Hepatitis C virus (HCV)-infected macrophages contain miR-29 that, when transferred to hepatocytes, trigger innate responses against the virus [64]. It was also shown that IFN-pulsed macrophages release vesicles that induce a late, but long-lasting, inhibition of HCV replication in hepatocytes [65]. In summary, these studies suggest that vesicles shed by human macrophages can enhance the immune response in recipient cell by delivering viral components, miRNAs, or interferon-stimulated genes able to restrict viral infection.

## 5. EVs from Macrophages and Parasitic Infections

The role of EVs from parasitized cells with Leishmania and Toxoplasma species, among other protozoan parasites, has been well studied in murine macrophages [87,88,89,90]. Concerning human macrophages, the studies are limited (Table 1). For instance, it was observed that EVs released from macrophages infected with *Toxoplasma gondii* transport PAMPs, which can stimulate pro-inflammatory responses in recipient macrophages via TLRs and MyD88 pathways [46]. Furthermore, Cestari and colleagues (2012) reported that vesicles from *Trypanosoma cruzi*-infected THP-1 cells inhibited, the complement-mediate parasite lysis through the inactivation of the classical and lectin pathway C3 convertases (C4b2a), indicating that T. cruzi can use EVs to inhibit the host immune response and, thus, enhance parasite survival and dissemination [66]. Recent findings showed that EVs released by T.cruzi-infected THP-1 cells activate TLR-2 and NF-kB pathway in recipient macrophages, resulting in enhanced production of pro-inflammatory cytokines (TNF-α, IL-6, and IL-1β) to maintain the inflammatory response in the course of infection [67]. These findings suggest that MΦ-EVs may enhance or impair host defense, thus controlling or facilitating the parasite survival and propagation.

## 6. Macrophage-Derived EVs and Fungal Infections

There are limited data on human macrophage-EVs in the context of fungal infections (Table 1). A quantitative proteomic study combined with bioinformatic analysis showed that the cargo of vesicles from monocyte-derived macrophages upon cell stimulation with linear (1,3)-β-glucan (curdlan) was highly enriched with integrins and other adhesion molecules. These EVs could induce host innate immune response via Dectin-1 receptor and contribute to spreading the infection by transferring danger signals directly to target cells [68]. Therefore, the functional roles of EVs from β-glucan-activated macrophages remain to be elucidated. Additional proteomic studies of particles secreted by macrophages infected with *Candida albicans* showed an increase number of proteins involved in cell signaling, cytoskeletal reorganization, and immune response [69]. In addition, THP-1-derived EVs, both from uninfected and Candida-infected macrophages, were able to induce similar effector functions in naïve macrophages by activating ERK and p38 kinases pathways, leading to the secretion of pro-inflammatory cytokines to control C. albicans infection [69]. Given the limited number of studies evaluating the possible role of EVs from human macrophages on fungal infections, more research is needed and encouraged to strengthen our knowledge about the impacts of vesicles secreted from these cells on the progression of diseases caused by pathogenic fungi.

## 7. Methods for Isolation and Characterization of EVs from Human Macrophages

At the same time that major advances were achieved in the understanding of the role of EVs in cellular communication and homeostasis, as well as of their clinical use as biomarkers or for therapeutic purposes, a large variety of technologies and strategies for isolation and characterization of extracellular vesicles were designed, developed, and improved [8,91,92]. Because comprehensive reviews of the available methods for EV analysis describing their working principles, improvements, and limitations were recently published elsewhere [13,93,94,95], here we will highlight the techniques more frequently used for studying EVs secreted by human macrophages (Table 2).

**Figure 2:**
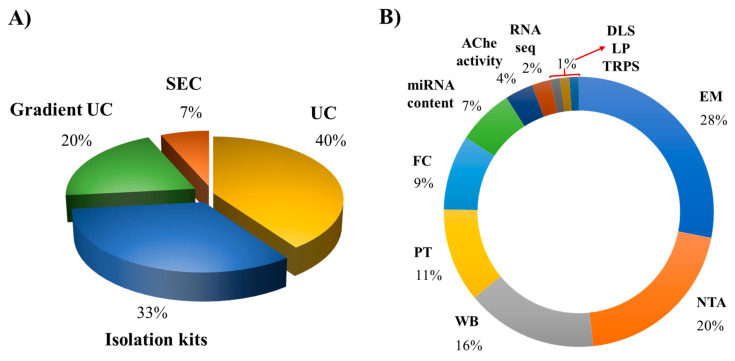
Pie chart of common technologies used for the separation and description of EVs released by human macrophages. (**A**) isolation and (**B**) characterization methods. Abbreviations: UC, ultracentrifugation; SEC, size-exclusion chromatography; EM, electron microscopy; NTA, nanoparticle tracking analysis; WB, western blotting; PT, proteomic analysis; FC, flow cytometer; AChe, acetylcholinesterase activity; DLS, dynamic light scattering; LP, lipidomic analysis; TRPS, tunable resistive pulse sensing.

To date, methods based on differential centrifugation (dUC) or ultracentrifugation (UC) have been used by approximately 40% of the works, followed by isolation kits using polymer-based precipitation (33%), sucrose or iodixanol gradient ultracentrifugation (20%), and finally the size-exclusion chromatography (SEC; 7%) (Figure 2). Each method presents advantages and shortcomings, and selecting the better method for a particular study depends on different factors, including the starting material of the cell culture, purity grade of cells, the volume of the cell conditioned medium, and the isolation purpose (e.g., research, therapeutic, or diagnostic use) [1].

We recently proposed a protocol based on sucrose density gradient ultracentrifugation (S-DGUC) with higher speeds (130,000× *g*) that allowed the separation of large amounts of small EVs released by monocyte-derived human primary macrophages enriched with vesicles from endosomal origin [96]. This work showed the method reproducibility and feasibility by using macrophage samples from different healthy donors to recover approximately 80% of a heterogeneous population of primary macrophage-derived small EVs. We performed this work taking into account the limited number of studies describing procedures for recovering small EVs from human primary macrophages, aiming to contribute with an improved method for research studying EVs from those cells [96]. To characterize MΦ-EVs, most researchers apply their physical (e.g., size, morphology, particle concentration) and molecular/biochemical properties (e.g., surface marker expression, nucleic acid, lipid, and protein contents) (Table 2). They report the use of electron microscopy (transmission, scanning or cryogenic electron microscopy), western blotting, nanoparticle tracking analysis, proteomic analysis, flow cytometer, acetylcholinesterase activity, dynamic light scattering, lipidomic analysis, and tunable resistive pulse sensing, achieving a usage percentage of 25%, 18%, 14%, 10%, 8%, 6%, 3% and 1%, respectively (Figure 2). The characterization techniques also present advantages and limitations [94]. Due to the minor differences in the physical properties and composition of the different EV subtypes, the combination of diverse techniques is required for qualitative and quantitative EVs characterization. In fact, the international society of extracellular vesicles (ISEV) recommends expanding the number of characterization methods to at least three or more independent techniques to describe heterogeneous populations of EVs [1]. The development and improvement of a large variety of advanced methods for EV analysis will be essential for the proper interpretation of results obtained in functional studies with EVs from different origins, potentially providing new opportunities for their applications in biomedical research and therapeutic purposes [97,98].

## 8. Macrophage-EVs as Diagnostic Markers for Infectious Diseases

Increasing evidence suggests the potential role of human macrophages for diagnostic and prognostic biomarkers during infectious diseases [99,100,101,102]. For instance, Lidofsky and colleagues demonstrated that soluble hemoglobin scavenger receptor (sCD163) from macrophages in patients infected with HIV-1 and HCV was positively associated with the severity of liver fibrosis from mild to moderate stage, with an Ishak fibrosis score up to 4, but not in established cirrhosis [103]. Moreover, the soluble form of the mannose receptor (sMR, sCD206) expressed by macrophages was significantly elevated in patients with sepsis and in those who died within 28 days. In addition, higher sensitivity of sMR was found to better predict mortality than other inflammatory markers such as procalcitonin (PCT), C-reactive protein (CRP), and sCD163 [104]. Serum expression levels of miR-145 from macrophages were observed to distinguishing Mtb-infected patients from healthy individuals and differentiating between active and latent tuberculosis cases [105]. Similarly, macrophages containing SARS-CoV-2 viral particles were found to express high levels of IL-6, and the presence of this inflammatory mediator was associated with severe depletion of lymphocytes from the spleen and lymph nodes in severe Coronavirus disease 2019 (COVID-19) patients [106].

In the last decade, EVs have attracted enormous research interest for their promising medical applications [77,107,108]. Vesicles may serve as disease diagnostic tools because they transport origin-specific subsets of nucleic acid (e.g., specifics miRNAs) and proteins that likely correlate to cell-type-associated functions [109,110]. These data and other studies quoted in this present review suggest that EVs secreted by infected human macrophages may carry miRNAs and/or protein signatures that closely reflect the associated clinical pathophysiology status in infectious diseases. Nonetheless, further studies using MΦ-EVs are necessary to understand and prove their potential use as diagnostic markers. Future investments on macrophage EVs-based tools could be a good strategy for diagnosing and prognosis of several human infectious diseases. Numerous advantages have been proposed for using EV-based diagnostics from other cellular models, such as reduction in patient pain, stress-free and low-cost alternatives compared with conventional techniques (e.g., excision biopsies or traditional needle biopsies) [111].

## 9. Concluding Remarks

**Figure 3:**
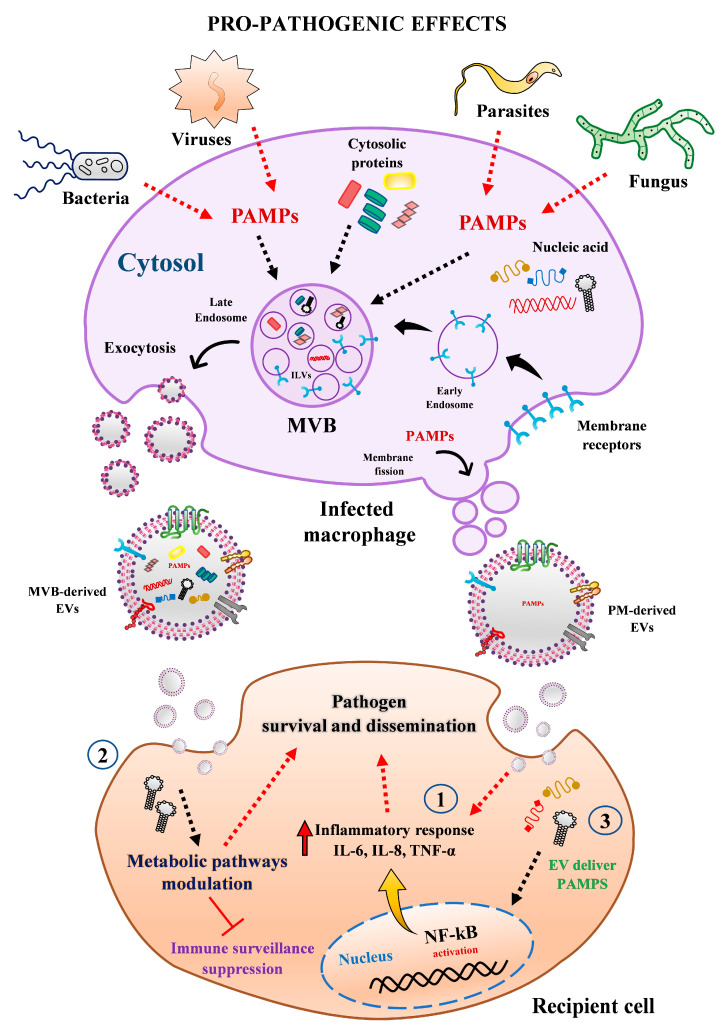
Pro-pathogenic effect mediated by EVs released from human macrophages. Macrophages infected with bacteria, virus, parasite, or fungi secreted heterogenous populations of EVs from different origin and sizes, which may favor pathogen growth and propagation through multiple mechanisms. For instance, (1) EVs from infected cells can trigger the secretion of pro-inflammatory cytokines (e.g., IL-6, IL-8, TNF-α) in recipient cells via activation of NF-kB pathway. (2) MΦ-EVs from infected cells can reprogram recipient cell metabolism pathways and modulated immune surveillance through transferring specific miRNAs. (3) EVs can be exploited by pathogens as alternative strategy to export microbial components (e.g., proteins, RNAs, miRNAs) to impair host defense and promote their infectivity and propagation. Abbreviations: MVBs, multivesicular bodies; PAMPs, pathogen-associated molecular patterns; PM, plasma membrane.

**Figure 4:**
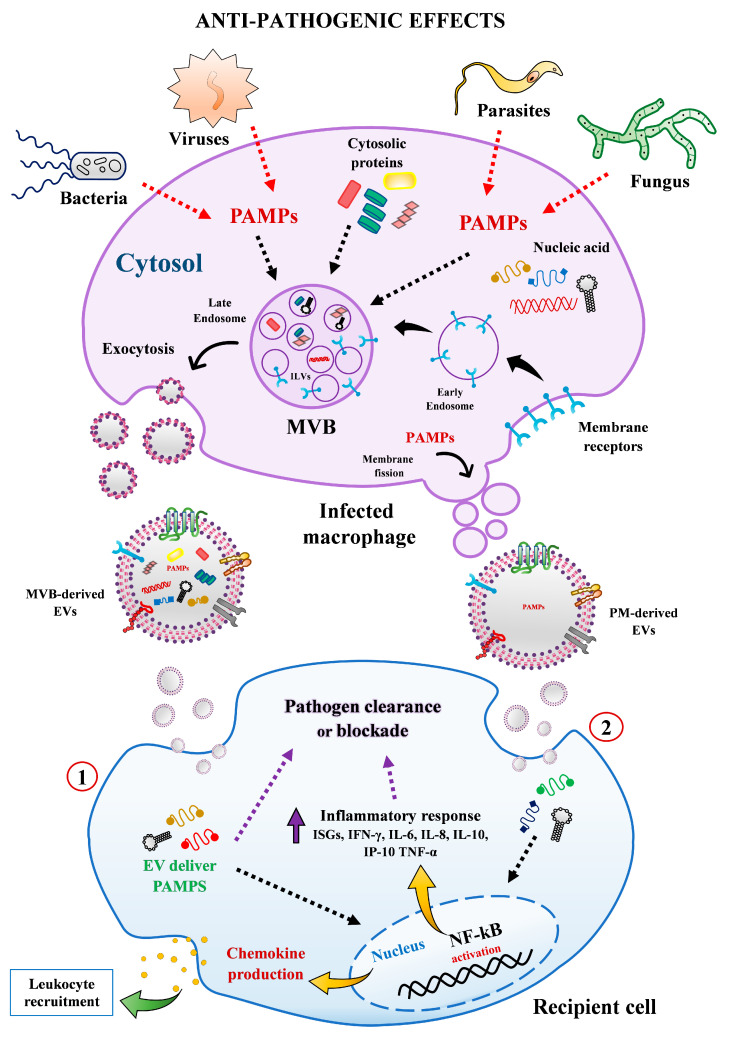
Anti-pathogenic effect mediated by EVs released from human macrophages. EVs secreted by infected macrophages may stimulate the clearance or blockade of pathogen growth in recipient cells by the following ways: (1) transporting PAMPs or pathogens antigens to enhance the expression of chemokines and recruitment of leukocytes to control the pathogen dissemination. (2) Transferring or stimulating proteins and molecules with anti-pathogenic effects, such as IFN-γ, IL-1β, IL-8, IL-6, IL-10, IP-10, TNF-α to diminish cell permissiveness to pathogen invasion. Abbreviations: MVBs, multivesicular bodies; PAMPs, pathogen-associated molecular patterns; PM, plasma membrane.

Extracellular vesicles from human macrophages have been on the central stage of many functional studies dealing with steady-state homeostasis or pathological conditions, especially in host-pathogen scenario. Although there is still much to be learned about their implication in the physio-pathogenesis of infectious disease, considerable knowledge has been gained in recent years. According to available evidence, EVs secreted by human macrophages may favor pathogen growth and dissemination in the following ways. (1) EVs from infected cells can trigger the secretion of pro-inflammatory cytokines in recipient cells via activation of specific cell signaling pathways (e.g., NF-kB). (2) MΦ-EVs from infected cells can reprogram recipient cell metabolism pathways and modulated immune surveillance through specific miRNA transference. (3) EVs can be exploited by pathogens as alternative strategy to export microbial components (e.g., proteins, RNAs, miRNAs) to impair host defense and promote infectivity and propagation (Figure 3). On the contrary, these EVs can reduce the replication of infectious agents by (1) transporting PAMPs or pathogens antigens to enhance the expression of chemokines and recruitment of leukocytes and stimulate the secretion of pro-inflammatory mediators to diminish cell permissiveness to pathogen invasion. (2) Transferring or stimulating in recipient cell the secretion of proteins and molecules with anti-pathogenic effects, such as IFN-γ, IL-1β, IL-8, IL-6, IL-10, IP-10, TNF-α (Figure 4). Overall, these studies indicate that EVs from human macrophages can either enhance or impair the host anti-pathogenic response.

The functional relevance of EVs from human macrophages during infection diseases is incompletely characterized. Future studies centering on their production, composition, diversity, and physiology may clarify various questions that remain open. Scientific efforts in the field may seek to develop advanced methodologies to allow full-scale recovery of particles, and to discriminate relevant subpopulations for the proper interpretation of EV-related studies. In summary, understanding the host–pathogens interactions in the context of human macrophage-EV-mediated cellular communication may provide new insights for future innovative therapeutic development based on the physiologic roles of extracellular vesicles.

## Figures and Tables

**Table 1:** Available studies * of EVs released by human macrophages during infectious diseases.

Pathogen	EV Effect on Pathogenesis	Source of EVs	Outcome in Recipient Cells	References
**BACTERIA**				
*Haemophilus influenzae*, *Moraxella catarrhalis*, *Streptococcus pneumoniae*, and *Pseudomonas aeruginosa*	Dual role	THP-1	Trigger and/or dampen immune responses against bacterial infection	[40]
*Escherichia coli*	Dual role	THP-1	Favor or inhibit bacterial replication	[41]
*Mycobacterium bovis* Bovis Bacillus Calmette–Guerin (BCG)	Enhancement	Primary monocyte-derived macrophage (MDM)	Increase bacterial replication and survival	[42]
*Treponema pallidum*	Enhancement	THP-1	Affect adhesion and permeability of human endothelial cells	[43]
	Enhancement	THP-1	Increase evasion of host immune response	[44]
*M. tuberculosis* (M. tb)	Enhancement	THP-1	Induce changes in EV protein composition to increased bacterial infection	[45]
*Mycobacterium tuberculosis*, *M. bovis* BCG, *Salmonella typhimurium*	Inhibition	THP-1	Enhance host immune response	[46]
*M. tuberculosis* (M. tb)	Inhibition	THP-1	Promote inflammation, intercellular communication, and cell migration to contain bacterial infection	[47]
	Inhibition	THP-1	Induce type I IFN response against M. tb infection	[48]
*Mycobacterium avium* sp. *paratuberculosis*	Inhibition	THP-1	Enhance host immune response	[49]
*M. avium*	Inhibition	THP-1	Enhance host immune response	[50]
*M. bovis* BCG	Inhibition	THP-1	Increase host innate immune response via endosomal TLR-7 activation	[51]
*Legionella pneumophila*	Inhibition	THP-1 or MDM	Stimulate immune response to control bacterial infection	[52]
*Salmonella enterica* serovar Typhimurium	Inhibition	THP-1	Enhance the release of pro-inflammatory mediators to control bacterial infection	[53]
**VIRUSES**				
HIV-1	Enhancement	MDM	Increase viral infection	[54]
	Enhancement	U937 or MDM	Increase HIV-1 reactivation in latent cellular reservoirs	[55]
	Enhancement	THP-1 or alveolar macrophages	Increase chronic immune activation through HIV vmiR88 and vmiR99	[56]
	Enhancement	MDM	Increase viral infection	[57]
	Enhancement	THP-1 or MDM	Affect epithelial barrier integrity and mitochondrial activity which contribute to immune dysfunctions against viral infection	[58]
	Enhancement	MDM	Promote hyperproliferation of pulmonary arterial smooth muscle cells	[59]
	Enhancement	MDM	Induce neuronal apoptosis and dysfunction through cathepsin B	[60]
H1N1	Enhancement	MDM or THP-1	Affect innate immune response in macrophages and DC against viral infection	[61]
DENV	Inhibition	U937	Induce activation of endothelial cells and promote endothelial barrier changes to increase proinflammatory response during infection	[62]
HBV	Inhibition	THP-1	Induce anti-HBV activity	[63]
HCV	Inhibition	MDM	Induce anti-HCV activity	[64]
	Inhibition	MDM or THP-1	Induce anti-HCV activity	[65]
**PARASITES**				
*Toxoplasma gondii*	Inhibition	THP-1	Enhance host immune response	[46]
*Trypanosoma cruzi*	Enhancement	THP-1	Inhibit innate immune response	[66]
	Undefined	THP-1	Undefined	[67]
**FUNGI**				
Fungal cell components: β-glucans	Inhibition	MDM	Activate innate immune system via Dectin-1 receptor	[68]
*Candida albicans*	Inhibition	THP-1	Enhance host immune response	[69]

Abbreviations: DENV, Dengue virus; HBV, Hepatitis B virus; HCV, Hepatitis C virus; HIV-1, human immunodeficiency viruses type I; HIN1, influenza A virus subtype H1N1; MDM, circulating human monocyte-derived macrophages. * PubMed, May 2021.

**Table 2:** Methods applied to separate and characterize EVs from human macrophages.

Source of EVs	Isolation Methods	Characterization Methods	References
**BACTERIA**			
THP-1	SEC	EM, FC, and TRPS	[40]
THP-1	Isolation kit	EM and WB	[41]
MDM	Isolation kit	EM, NTA, and miRNA content	[42]
THP-1	Isolation kit	EM, NTA, and WB	[43]
THP-1	Isolation kit	EM, NTA, miRNA content, and WB	[44]
THP-1	Isolation kit	EM, NTA, PT, and WB	[45]
THP-1	UC and sucrose density gradient separation	EM, FC, and WB	[46]
THP-1	UC	EM, FC, and PT	[47]
THP-1	UC	PT	[48]
THP-1	UC	EM, FC, and PT	[49]
THP-1	Isolation kit	EM	[50]
MDM	UC	EM, NTA, RNA-seq, and WB	[51]
THP-1	UC	EM, NTA, and WB	[52]
THP-1	UC and iodixanol density gradient separation	EM, NTA, PT, and WB	[53]
**VIRUSES**			
MDM	UC and sucrose density gradient separation	EM, LP, and PT	[54]
U937 or MDM	UC and iodixanol density gradient separation	AChE activity and FC	[55]
THP-1 or primary human alveolar macrophages	Isolation kit	miRNA content and RNA-seq	[56]
MDM	UC and iodixanol density gradient separation	AChE activity, miRNA content, and WB	[57]
THP-1 or MDM	UC	EM, NTA, and WB	[58]
MDM	UC	EM, NTA, and WB	[59]
MDM	Isolation kit	NTA and WB	[60]
MDM or THP-1	Isolation kit	NTA and miRNA content	[61]
U937	UC and sucrose density gradient separation	AChE activity, EM, miRNA content, PT, and WB	[62]
THP-1	UC	EM, FC, and WB	[63]
THP-1	Isolation kit	EM and WB	[64]
MDM or THP-1	UC	NTA, EM, FC, and WB	[65]
**PARASITES**			
THP-1	UC and sucrose density gradient separation	EM, FC, and WB	[46]
THP-1	UC	EM and FC	[66]
THP-1	SEC	EM, NTA, PT, and WB	[67]
**FUNGI**			
MDM	UC	EM, NTA, PT, and WB	[68]
THP-1	UC	DLS, EM, and PT	[69]

Abbreviations: UC, ultracentrifugation; SEC, size-exclusion chromatography; EM, electron microscopy; NTA, nanoparticle tracking analysis; WB, western blotting; PT, proteomic analysis; FC, flow cytometer; AChe, acetylcholinesterase activity; DLS, dynamic light scattering; LP, lipidomic analysis; TRPS, tunable resistive pulse sensing.

## Data Availability

The authors declare that all data presented in this manuscript are fully available.
